# Preliminary MRI-based investigation of characteristics and prognosis of knee bone marrow edema in children with juvenile idiopathic arthritis

**DOI:** 10.1007/s10067-022-06085-3

**Published:** 2022-02-09

**Authors:** Yang Yang, Xinyu Yuan, Xinning Wang, Ran Tao, Tao Jiang

**Affiliations:** 1 Department of Radiology, Beijing Chao-Yang Hospital, Capital Medical University, Beijing, China; 2 Department of Radiology, The Affiliated Children’s Hospital, Capital Institute of Pediatrics, Beijing, China; 3 Department of Rheumatology and Immunology, The Affiliated Children’s Hospital, Capital Institute of Pediatrics, Beijing, China

**Keywords:** Bone marrow edema, Juvenile idiopathic arthritis, Knee joint, Magnetic resonance imaging

## Abstract

**Introduction:**

Bone marrow edema (BME) is one of the main imaging characteristics of juvenile idiopathic arthritis (JIA) in children and rheumatoid arthritis (RA) in adult. Previous studies have shown that BME occurred in approximately 64% of adults with RA and was a key predictor of poor prognosis. But BME with JIA has not been of great concern. Therefore, we evaluated the prevalence, characteristics, and prognosis of knee joint BME in children with JIA.

**Methods:**

In this retrospective study, we included children with JIA and knee joint involvement from January 2017 to December 2019. BME was evaluated according to the Juvenile Arthritis MRI Scoring system. Clinical characteristics were compared between the BME group and the non-BME group. The characteristics and prognosis of the BME were observed.

**Results:**

A total of 128 children with 136 knee joint MRI data were identified, with 37 knee joints (27.2%) having BME. BME has positive correlation with synovial hypertrophy (*Rs* = 0.562, *p* = 0.019). There were significant differences in age (*p* = 0.010) and disease duration (*p* = 0.013) between the BME and non-BME groups. BME was found to be more common in older children and the patients with long duration of disease. Locations with BME were the lateral tibial plateau (17/37, 45.9%), the lateral weight-bearing femur (16/37, 43.2%), the medial tibial plateau and the medial femoral condyle (both with 15/37, 40.5%), and the medial weight-bearing femur (12/37, 32.4%). The lateral femoral condyle and both the lateral and medial sides of the patella were rarely involved. Of the 15 BME joints with the MRI follow-up data (interval 6.5 ± 3.0 months), the lesions disappeared or improved within 12 months after the treatments in 13 (86.7%) joints.

**Conclusions:**

The prevalence of knee BME in JIA was 27.2%. There was positive correlation between BME and synovial hypertrophy. Older children and children with long disease duration had a higher risk for BME, which was commonly a late presentation and more likely involved the weight-bearing surfaces of the joint. The overall prognosis was satisfactory after the standard treatments.

**Table:** 

**Key Points** *• To the best of our knowledge, this paper is the first one to investigate the MRI manifestation in JIA focus on knee BME sign.*

## Introduction

Juvenile idiopathic arthritis (JIA) is a common pediatric articular disorder. Early diagnosis and timely interventions can prevent irreversible damage to the joint, thereby improving the prognosis. The knee is the most commonly involved joint, and is considered to be the most appropriate joint in JIA for evaluation of outcome with magnetic resonance imaging (MRI) [1, 2].

JIA is characterized by prolonged synovial inflammation that can lead to destruction of joints; the main imaging features include synovial thickening, joint effusion, and bone marrow edema (BME) [3]. Previous studies have shown that synovial inflammation and joint effusion in JIA are closely related to clinical arthritis activity, and the prognosis is good [4, 5]. The study on BME as an important manifestation of early bone involvement is at present lacking [6]. The studies of adults with rheumatoid arthritis (RA) have shown that BME occurred in approximately 64% [7] and was a key predictor of poor prognosis [8, 9]. The prevalence of bone destruction secondary to the BME in RA patients ranged from 34 to 68% [10]. Children have rapid skeletal growth and development. What are the characteristics of BME caused by JIA? There are challenges for pediatric rheumatologists and radiologists. However, there have been limited studies to investigate the BME in JIA; we evaluated the prevalence, characteristics, and prognosis of knee joint BME in children with JIA in the present study.

## Methods

### Study design and participants

We retrospectively identified children with JIA diagnosed at the Department of Rheumatology and Immunology of our hospital between January 2017 and December 2019. The study protocol was approved by the hospital ethics committee.

The patients met all the following inclusion criteria: (1) children with JIA diagnosed based on the criteria from the International League of Associations for Rheumatology (ILAR) [11]; (2) knee arthritis clinically; (3) knee MRI was performed before treatment. Children with history of hypermobility or trauma, or other systemic disease were excluded. We collected clinical information of the included patients, including sex, age, disease duration, and JIA type.

### Magnetic resonance imaging examination

A GE Signa 1.5-T scanner (General Electric, MA, USA) was used to examine the knee joint. The children were placed in the supine position with the knee joint lying centrally in the magnetic field in the dedicated knee coil. Children who could not cooperate were sedated with oral chloral hydrate (0.5 mL/kg). The sagittal T1WI/T2WI/STIR (short tau inversion recovery) sequences, axial STIR, and sagittal and coronal fat-suppressed T1WI scans after intravenous injection of Gd-DTPA (0.2 mL/kg) were performed.

### Image analysis

BME was evaluated in accordance with the latest version of the Juvenile Arthritis MRI Scoring (JAMRIS) system, which has been validated and described before in detail [3, 12]. All patients underwent complete imaging assessments. BME was defined as ill-defined lesions within the trabecular bone, presenting high signal intensity on T2-weighted images and low signal intensity on T1-weighted images. The presence of bone marrow changes was scored in eight anatomical regions, including medial and lateral patella on the axial view, medial and lateral femoral condyle, and the medial and lateral weight-bearing surfaces of the femur. The tibia was divided into two regions: the medial and lateral tibial plateau. Bone marrow changes suggestive of BME were scored semi-quantitatively based on the subjectively estimated percentage of the involved bone volume at each site as follows: grade 0, none; grade 1, < 10% of the whole bone volume; grade 2, 10–25% of the whole bone volume; grade 3, > 25% of the whole bone volume. The total score could be in the range between 0 and 24.

After receiving appropriate training in JAMRIS, two experienced pediatric radiologists independently blindly evaluated the MRI data from the enrolled children, and their mean score was taken as the final score.

In addition, relationship between BME and synovial thickening, the basic pathological change of JIA, was explored.

### Statistical analysis

Statistical analyses were performed in SPSS (version 25.0, IBM, NY, USA). Categorical data were presented as percentages. Continuous data were presented as mean ± standard deviation or median with interquartile ranges, depending on the results of the normality test. The linear correlation was tested using the Spearman rho. Significance was classified as follows: *Rs* < 0.40, poor; ≥ 0.40–0.60, moderate; > 0.60–0.80, substantial; and > 0.80, good correlation. Kappa statistic was used to test the inter-rater agreement between two radiologists. Based on the MRI score, all of the patients were classified either into the BME group or non-BME group. Clinical characteristics were compared between the two groups by the *t* test, nonparametric test, or chi-square test, as appropriate. A *p* value < 0.05 was considered statistically significant.

## Results

A total of 128 JIA children, with 136 knee MRI images, were included in the present study. There were 61 boys and 67 girls, with the mean age of 6.7 ± 3.9 years. For 15 children, follow-up knee MRI data were available (mean follow-up interval 6.5 ± 3.0 months).

There was high agreement on the score of BME evaluations between the two radiologists (kappa = 0.81, *p* < 0.001). Of the 136 knee joints that received MRI examinations during the initial hospital visits, 32 showed BME. Another five knee joints showed BME in the follow-up MRI examinations during the treatments. Therefore, a total of 37 knee joints had BME during the observation period (37/136, 27.2%). And BME was accompanied by synovitis in all cases. There were significant correlations between total BME grade and synovial hypertrophy (*Rs* = 0.562, *p* = 0.019).

Baseline comparisons between the BME and non-BME groups are shown in Table 1. There were no significant differences in sex and the types of JIA between the two groups. There were significant differences in age (*t* = 2.631, *p* = 0.010) and the duration of disease (*z* =  − 5.782, *p* = 0.013) between the two groups. BME was found to be more common in older children and the patients with long duration of disease.

**Table 1:** Clinical characteristics of 128 children with juvenile idiopathic arthritis

Variables	BME group	Non-BME group	Statistic	*p*
Number of children/number of affected joints	36/37	92/99		
Sex, male:female	14:22	45:47	*z* = − 1.019	0.308
Age, years, mean ± standard deviation	8.1 ± 3.7	6.1 ± 3.9	*t* = 2.631	0.010*
Disease duration, months, median (interquartile ranges)	9 (3, 22)	4 (2, 18)	*z* = − 5.782	0.013*
Types, *N*			*z* = − 0.078	0.938
Oligoarthritis	18	50		
Polyarticular arthritis (negative RF)	6	5		
Polyarticular arthritis (positive RF)	2	2		
Systemic	5	27		
Enthesitis-related arthritis	5	8		

*BME*, bone marrow edema; *RF*, rheumatoid factor.

*t* refers to *t* test. *z* refers to nonparametric test. *p < 0.05.

**Fig. 1:**
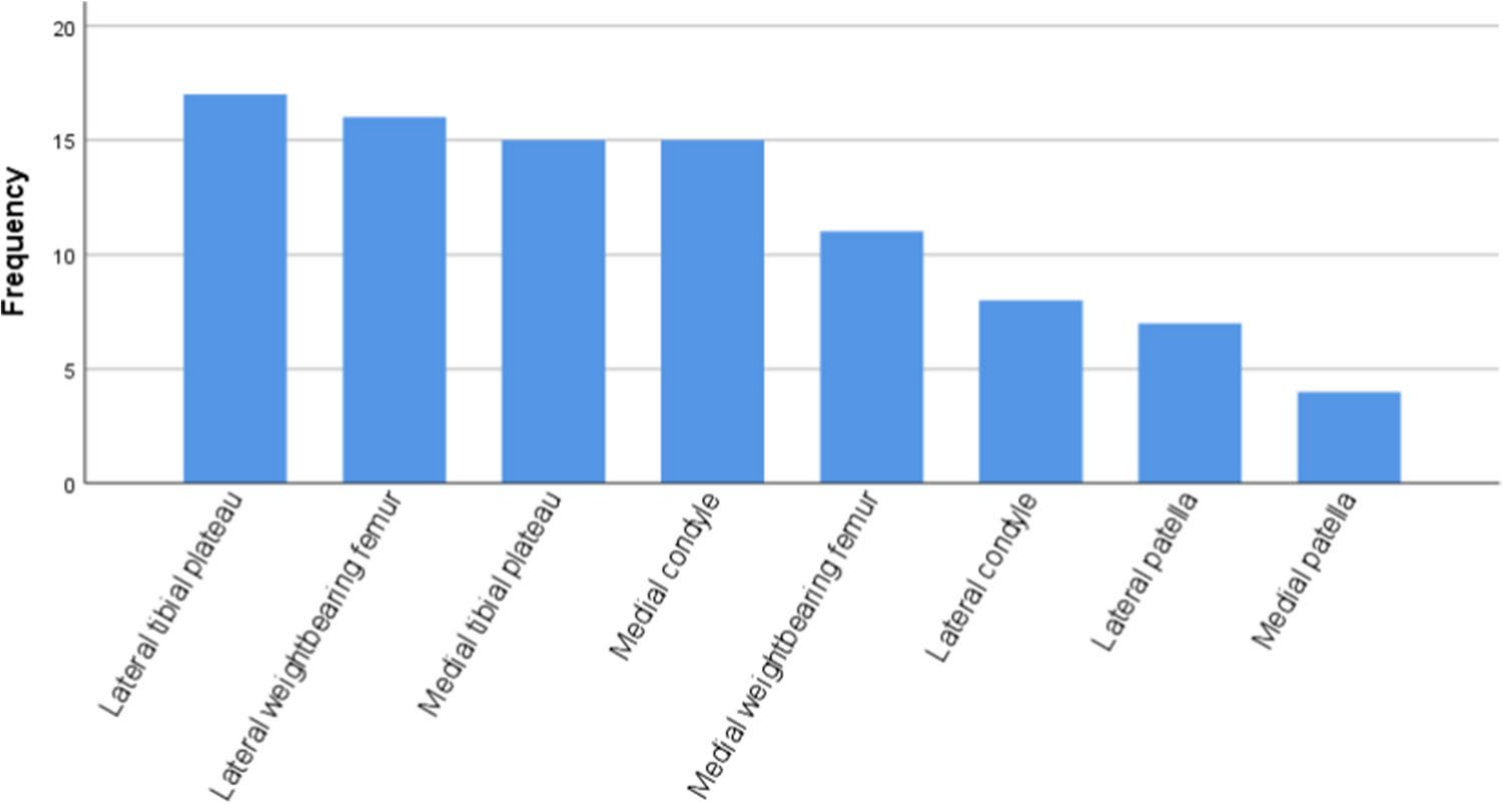
Frequencies of involvements at different locations in the knee joint in children with JIA

**Fig. 2:**
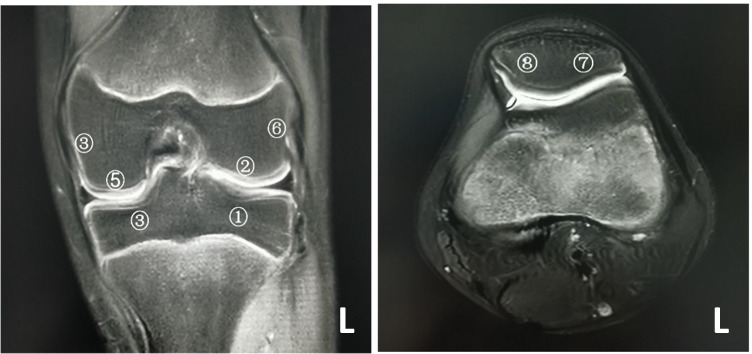
Locations frequently affected by bone marrow edema identified in the study

**Fig. 3:**
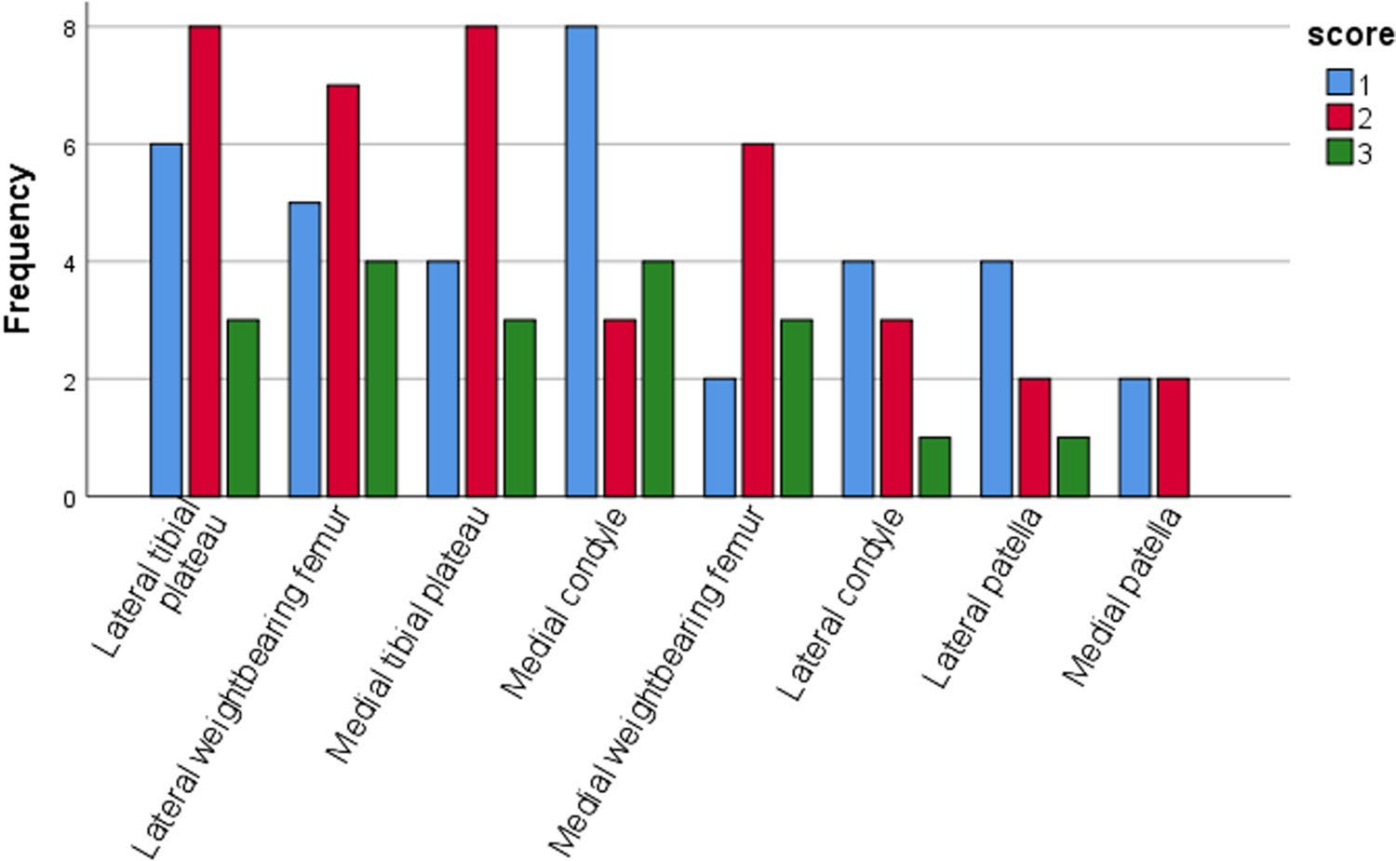
Degrees of involvements at different locations in the knee joint in children with JIA

Based on the JAMRIS system, the 37 knee joints with BME had the lesions to varying degrees at eight locations. The frequencies of lesions at each location are shown in Fig. 1. The most frequently involved locations were the lateral tibial plateau (17/37, 45.9%) and the lateral weight-bearing femur (16/37, 43.2%), followed by the medial tibial plateau (15/37, 40.5%) and the medial femoral condyle (15/37, 40.5%), and the medial weight-bearing femur (12/37, 32.4%). The lateral femoral condyle and the lateral and medial sides of the patella were rarely involved. Locations frequently affected by BME in the descending order are shown in Fig. 2. It is noteworthy that the medial femoral condyle usually had a mild involvement with a major score of 1 (Fig. 3).

Among 37 knees with BME, 62.2% of the knees had lesions at multiple locations. Among 14 knees (37.8%) with signs of BME in only one location, BME was located at the medial femoral condyle in nine cases, at the lateral tibial plateau in three cases, and at the lateral weight-bearing femur in two cases.

**Fig. 4:**
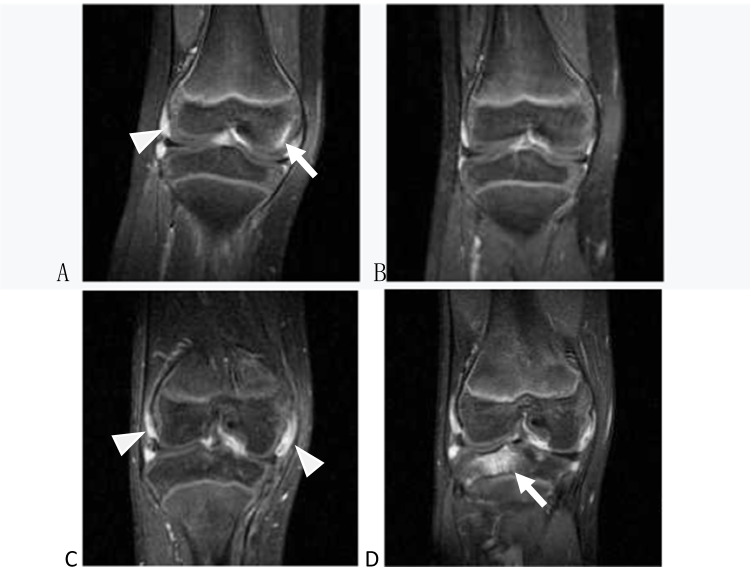
**A**, **B** 7-year-old girl, coronal T1-W FS Gd-enhanced MRI. **A** BME in the medial femoral condyle (arrow). Note the accompanying synovitis (arrowhead). **B** BME disappeared at the follow-up visit 6 months after standard treatment. **C**, **D** 11-year-old boy, coronal T1-W FS Gd-enhanced MRI. **C** No BME was visible at the initial MRI examination, but the synovitis was clear (arrowhead). **D** After 10 months with reduced dosage even withdrawal, BME appeared in the lateral tibial plateau (arrow)

Among 37 knees with BME, 15 underwent MRI follow-up re-examination during the treatment, with a follow-up interval of 6.5 ± 3.0 months. Within the 12-month follow-up, BME signs disappeared in ten knees, improved in three knees, and progressed in two knees (Table 2, Fig. 4). The rate of BME disappearance and improvement within 12 months was 86.7% (13/15), while the rate of progression was 13.3% (2/15). All of the 13 patients with improved BME were treated with traditional immunosuppressants, including methotrexate and leflunomide, seven of them combined with NSAIDs, and six patients combined with biological agents (infliximab or tocilizumab).

**Table 2:** General information and BME changes of 15 patients with MRI follow-up

ID	Gender	Age (year)	Follow-up interval (month)	BME locations and scores (pre value/post value)							
				LTP	LWBF	MTP	MC	MWBF	LC	LP	MP
1	F	7	4				1/0				
2	M	8	3			2/0					
3	F	10	10	1/0			1/0				
4	F	5	7				1/0				
5	F	5	9		1/0			1/0			
6	F	2	4				1/0				
7	F	7	7			1/0					
8	F	9	3	2/0	2/0	2/0		2/0			
9	M	11	6	2/0		2/0	1/0		1/0	2/0	2/0
10	F	13	3			1/0					
11	M	11	7	2/1		2/1	1/0				
12	F	4	6		2/1	1/0		3/1			
13	F	8	6		3/3		3/2	3/3	3/0		
14	F	3	12		0/2						
15	F	11	12	0/2		0/1	0/1		0/1		

*Age* age of onset, *LTP* lateral tibial plateau, *LWBF* lateral weight-bearing femur, *MTP* medial tibial plateau, *MC* medial condyle, *MWBF* medial weight-bearing femur, *LC* lateral condyle, *LP* lateral patella, *MP* medial patella.

## Discussion

JIA refers to a heterogeneous group of conditions with onset under the age of 16 years with unknown etiology and persistence of symptoms for over 6 weeks [13]. BME is an important sign common in adults with RA and children with JIA. Histological studies on BME in RA patients showed local inflammation with increased vascularity. The osteoclastic activity was closely related to the local lymphocytic infiltration, which could lead to a poor prognosis [14, 15]. In recent years, with the widespread use of MRI, joint BME is often identified in children with JIA. In the present study, the prevalence of BME in JIA was 27.2%, which was lower than that reported in adult RA (64%) [7]. BME was accompanied by synovitis in all joints, there was no BME finding without synovitis in this study, and there was positive relationship between them. This finding is consistent with the traditional pathogenesis hypothesis for JIA, which think inflammation is from synovitis to cartilage damage to bone erosion, synovitis increased over time result in BME, synovitis is the precursor to bone involvement [16, 17].

The results of our study showed that the BME and non-BME groups had comparable distributions of sex and the types of JIA. This might be due to the fact that JIA children with different age and types of JIA had similar pathological changes of the involved joints, which resulted in similar presentations in the MRI images. BME was more likely to be found in older children, which might be related to the increased activity and knee joint loading in older children. The median duration of disease in the BME group was 9 months, which was significantly longer than that in the non-BME group (4 months), suggesting that BME might not happen during the early JIA development but rather occur gradually with the progression of JIA. This was different from the adult RA where BME was reported more frequently in the early disease development [7, 8].

The present study showed that most of the knee joints with BME (62.2%) showed simultaneous lesions in multiple locations. More lesions were observed in the weight-bearing surfaces and fewer lesions were found in the non-weight-bearing surfaces and patella. The most frequently and severely affected locations in the knee joint were the lateral tibial plateau and the lateral weight-bearing surface of the femur (lateral tibiofemoral joint, LTJ). LTJ was also the common location when a single BME lesion was identified in the knee joint. The possible reason for the frequent involvements of the LTJ was the physiological knee valgus in children more than 2 years old from the abnormal lower extremity force line due to weak joint capsule and ligament, unstable joints, and underdeveloped muscles [18]. The valgus stress line could place more pressure on the lateral surface of the tibiofemoral joint, which could result in BME. Therefore, reduction of weight-bearing in the lower extremities and correction of the abnormal force line might decrease the risk of BME. In addition to the weight-bearing surfaces, the medial femoral condyle was also frequently affected by BME. Nine out of 12 BME knee joints with single lesions affected the medial femoral condyle. This location was not involved in the weight-bearing. Its lesion could not be explained by the stress from the force line. Besides, our study found that BME at the medial femoral condyle was less severe and had a better prognosis than BME at the weight-bearing surfaces in the knee joint. A previous study found that, compared with the lateral femoral condyle, the medial femoral condyle was more likely to have a higher degree of intrachondral ossifications with irregular shapes and uneven signals in the MRI images [19]. We presume that there would be a potential relationship between BME at the medial femoral condyle and altered blood supply during the developmental intrachondral ossifications. The exact mechanism requires further investigations.

The progression, chance of recovery, and risk of disability caused by BME in JIA children are the major concerns for the clinicians and parents. In the 15 knee joints displaying BME, for which the follow-up MRI studies were available, the lesions completely disappeared or improved within 12 months after standard treatments in 86.7% of cases, and no bone destruction or developmental disability occurred. This promising result may suggest significantly better prognosis of BME in childhood JIA compared with adult RA. Two children with poor recoveries of BME had poor compliance with the treatments, which might be responsible for their disease progression.

Our study had several limitations. First, we did not include children without JIA as a control group. Pro. Hemke had made a research on the normal appearance of knee in healthy children and reported that physiological changes similar to BME could be observed in the apex patellae in healthy children, but without other accompanying abnormalities [20]. Our data showed that all the BME change accompanied by synovial hyperplasia, so we think there was not enough evidence to take it as physiological change. Next, these children were followed up at different timepoints after the treatments. The exact time period required for the improvement or progression of BME was not clear. Third, we included a small sample size with a short follow-up period. The retrospective study design could also bring biases into the analysis. Finally, factors that could affect the BME prognosis and its possible recurrence were not investigated due to the small sample size. A prospective study with a large sample size should be conducted for further empirical support of the current findings.

## Conclusions

The prevalence of knee BME in childhood JIA was lower than that in adult RA from literatures. There was positive correlation between BME and synovial hypertrophy. Older children and children with long disease duration had a higher risk for BME. BME frequently presented as a late finding and more likely involved the weight-bearing surface of the knee. Most of the JIA children had various degrees of improvement and satisfactory outcomes after standard treatments.

## Data Availability

All data generated or analyzed during this study are included in this article.

## Abbreviations

BME: Bone marrow edema
JIA: Juvenile idiopathic arthritis
RA: Rheumatoid arthritis
MRI: Magnetic resonance imaging
ILAR: League of Associations for Rheumatology
JAMRIS: Juvenile Arthritis MRI Scoring
LTJ: Lateral tibiofemoral joint
